# Reactive Infectious Mucocutaneous Eruption Following a Respiratory Infection in an Adolescent: A Case Report

**DOI:** 10.7759/cureus.115443

**Published:** 2026-08-30

**Authors:** Final G Patel, Omeka Bhatia, Natalie Hogan, Michael Bossak

**Affiliations:** 1 School of Medicine, Mercer University School of Medicine, Savannah, USA; 2 Department of Pediatrics, Willett Children's Hospital of Savannah, Memorial Health University Medical Center, Savannah, USA

**Keywords:** mucositis, mycoplasma pneumoniae, pediatric dermatology, pediatric dermatology emergency, pneumonia, reactive infectious mucocutaneous eruption

## Abstract

Reactive infectious mucocutaneous eruption (RIME) is a recently defined mucocutaneous manifestation associated with respiratory infections, most commonly reported secondary to* Mycoplasma pneumoniae*. The core diagnostic criteria of RIME involve greater than or equal to two mucosal areas with erosive mucositis, minimal cutaneous lesions, a recent infectious prodrome, and exclusion of drug-induced etiologies. RIME presents with acute mucositis involving the oral, ocular, and genital mucosa, with presentations similar to other mucocutaneous disorders such as Stevens-Johnson syndrome (SJS), erythema multiforme (EM), and multisystem inflammatory syndrome in children (MIS-C) secondary to SARS-CoV-2 infection.

This case report describes a unique presentation of RIME secondary to a *Mycoplasma* infection, highlighting diagnostic challenges and therapeutic approaches.

A 16-year-old male, previously healthy with no significant past medical history, was accepted for transfer to our hospital for evaluation of persistent fever, worsening left lower lobe pneumonia, oral mucositis, and conjunctivitis. On examination, extensive erythema and inflammation throughout the oral cavity, exudative pharyngitis, clear ocular discharge with conjunctival injection, and sloughing of the scrotal skin were noted.

The patient's medical history and clinical examination raised initial suspicion of Kawasaki disease or possible MIS-C. Lab studies showed elevated CRP, ESR, and B-type natriuretic peptide (BNP), indicating extensive systemic inflammation; however, the PCR was negative for respiratory syncytial virus (RSV), influenza A and B, and SARS-CoV-2. Further investigations revealed a positive Group A *Streptococcus* PCR on throat swab, felt to be colonization, and elevated *Mycoplasma pneumoniae *IgG titers suggested previous or recent exposure; however, acute infection could not be confirmed due to negative PCR and IgM.

The patient improved over the following several days after initiation of azithromycin, continuation of clindamycin, and wound care for scrotal and oral mucosal lesions.

Differentiating RIME from other mucocutaneous disorders is vital to prescribing proper treatment and preventing further systemic effects. This case underscores the importance of considering *Mycoplasma* as a potential etiology in patients presenting with mucocutaneous lesions and conjunctivitis following a previous respiratory infection.

## Introduction

*Mycoplasma pneumoniae* is a common cause of community-acquired pneumonia in children and is associated with various extrapulmonary manifestations, including mucocutaneous eruptions [1]. Reactive infectious mucocutaneous eruption (RIME) includes reactions to *Mycoplasma pneumoniae* and a broader range of infectious agents like SARS-CoV-2, influenza A, and rhinovirus, prompting a nomenclature shift to RIME [2].

RIME is characterized by severe mucosal involvement, often affecting oral, ocular, and genital mucosa [1-3]. It is a phenomenon typically observed in younger populations, mainly children and adolescents [2]. While diverse, the clinical presentation of RIME is distinct from that of other mucocutaneous conditions, such as Kawasaki disease and multisystem inflammatory syndrome in children (MIS-C), underscoring the importance of accurate diagnosis [3].

RIME represents a rare and challenging diagnosis, often requiring a high index of suspicion and a multidisciplinary approach for management. This report details a complex case of RIME following a respiratory illness in which *Mycoplasma pneumoniae* and influenza B were considered as possible triggers, highlighting its diagnostic intricacies and therapeutic interventions.

## Case presentation

A previously healthy 16-year-old male patient presented to an urgent care facility with cold-like symptoms, including cough, congestion, and emesis that began three days prior. At the urgent care facility, he tested positive for influenza B. He was prescribed prednisone, fexofenadine, and guaifenesin. Despite initial treatment with prednisone, fexofenadine, and guaifenesin, his condition deteriorated. He subsequently presented to the emergency department (ED) two days later with a fever of 101.5°F and symptoms indicative of strep throat and left lower lobe (LLL) pneumonia, the latter confirmed by chest X-ray. Laboratory tests revealed a positive Group A *Streptococcus* PCR, systemic inflammation with elevated WBC count (15.6 × 10⁹/L), CRP (16 mg/dL), and ESR (16 mm/hour), alongside coagulation abnormalities (prothrombin time of 16.2 seconds and partial thromboplastin time of 31.5 seconds), but normal electrolytes and negative Monospot and blood cultures. Treatment at this stage included fluid boluses, pain medications, and clindamycin. Two days following, the patient's condition worsened, with new symptoms of conjunctivitis, fever, and severe oral mucosal involvement, leading to a transfer to our facility under suspicion of Kawasaki disease or MIS-C.

Upon transfer to our ED, the patient was febrile (102.7°F) and tachycardic (heart rate (HR): 120), with a blood pressure of 138/76, respiratory rate of 22, and SpO₂ of 93% on room air. A comprehensive physical examination revealed decreased LLL breath sounds, extensive erythema, inflammation throughout the oral cavity, exudative pharyngitis, bilateral clear eye discharge with conjunctival injection, and sloughing of the scrotal skin (Figure 1). Musculocutaneous physical exam was negative for target lesions, bullae, and dusky macules. 

**Figure 1:**
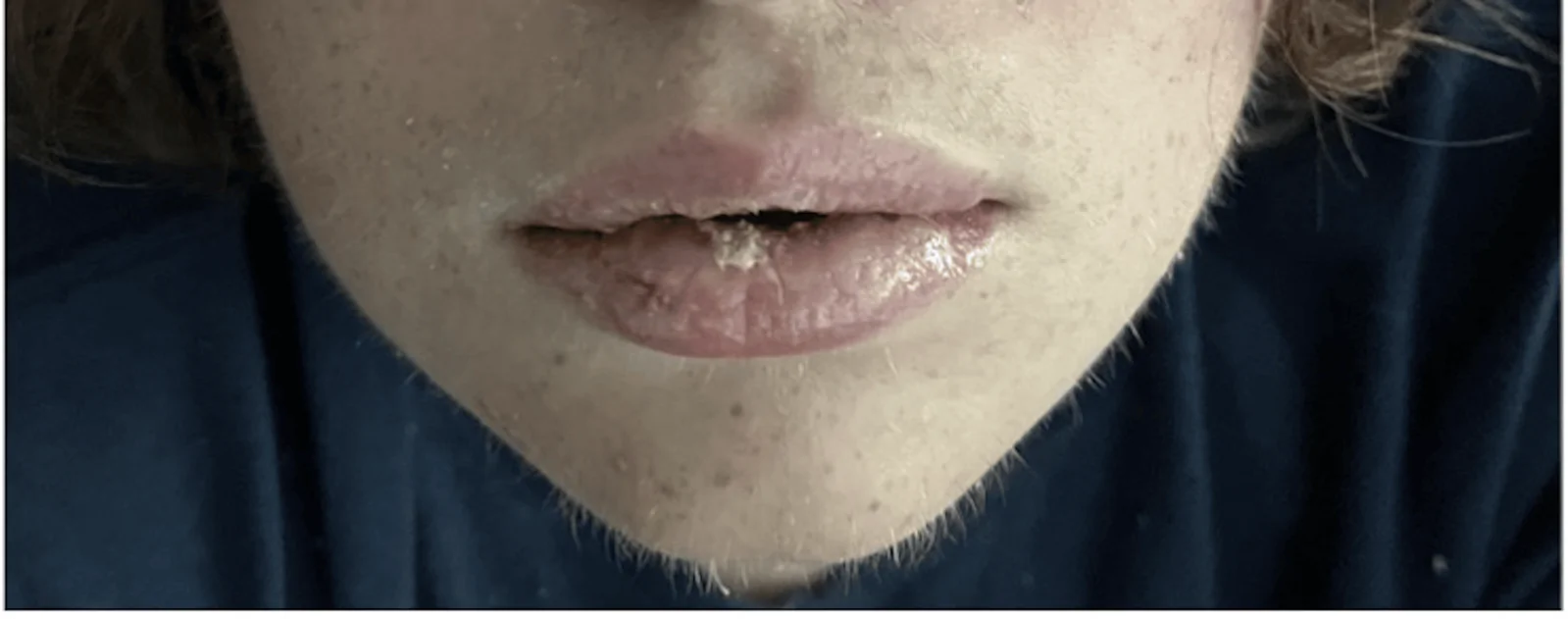
Oral mucositis and lip crusting on admission

As the oral lesions and tongue swelling progressed, the patient was unable to eat and was primarily consuming liquids as tolerated.

Chest X-rays were concerning for LLL pneumonia, and an extended respiratory panel was negative for respiratory syncytial virus (RSV), influenza A and B, and SARS-CoV-2. On admission, a full laboratory workup was obtained, with the pertinent results being shown in Table 1. Group A *Streptococcus* was identified in the throat, guiding continued clindamycin therapy. The patient was admitted to the pediatric ICU for further management.

**Table 1:** Initial laboratory results on admission

Laboratory Test	Patient's Value	Pediatric Normal Values	Interpretation
CRP (mg/dL)	12.8	<1.0 mg/dL	Elevated, indicates inflammation
BNP (pg/mL)	130	<125 pg/mL	Equivocal
D-dimer (ng/mL)	1451	<519 ng/mL	Elevated, suggests coagulopathy
Fibrinogen (mg/dL)	523	197-442 mg/dL	Elevated, indicates acute phase reactant
WBC (K/uL)	14.3	4.5-13.5 K/uL	Elevated, indicates infection or inflammation
RBC (M/uL)	4.06	4.5-5.3 M/uL	Low, may indicate anemia
Hemoglobin (g/dL)	11.7	13-16 g/dL	Low, may indicate anemia
Monocytes (%)	13.4	1-8 %	Elevated, indicates infection or inflammation
Group A *Streptococcus* (PCR)	Positive	Not detected	Indicates bacterial colonization/infection
Respiratory expanded screen with *Bordetella* (PCR)	Not detected	Not detected	Negative for viral respiratory infection and *Bordetella* infection

Due to concerns of disseminated gonococcal infection with gonococcal pharyngitis, STD testing was obtained along with repeat laboratory tests (Table 2). An echocardiogram was ordered, and the results were normal.

**Table 2:** Repeat laboratory values 2 days post-admission

Laboratory Test	Patient's Value	Pediatric Normal Values	Interpretation
CRP (mg/dL)	12.4	<1.0 mg/dL	Elevated, indicates persistent inflammation
BNP (pg/mL)	547	<125 pg/mL	Significantly elevated, may indicate increased cardiac strain
*Neisseria gonorrhoeae* (PCR)	Not detected	Not detected	Negative, no infection detected
*Chlamydia trachomatis* (PCR)	Not detected	Not detected	Negative, no infection detected
RPR serology	Non-reactive	Non-reactive	Negative, no syphilis detected
HIV-1 and HIV-2 antibodies	Non-reactive	Non-reactive	Negative, no HIV infection detected

Despite continuation of clindamycin and supportive care, oral mucositis, urogenital lesions, and conjunctivitis persisted (Figure 2), prompting consultations with Ophthalmology and Wound Care for targeted interventions, which included Polymyxin B and trimethoprim eye drops and comprehensive mucosal management with analgesia, debridement, and specific topical treatments (Aquaphor, bacitracin, and nystatin for scrotal candidiasis) (Figures 3-4).

**Figure 2:**
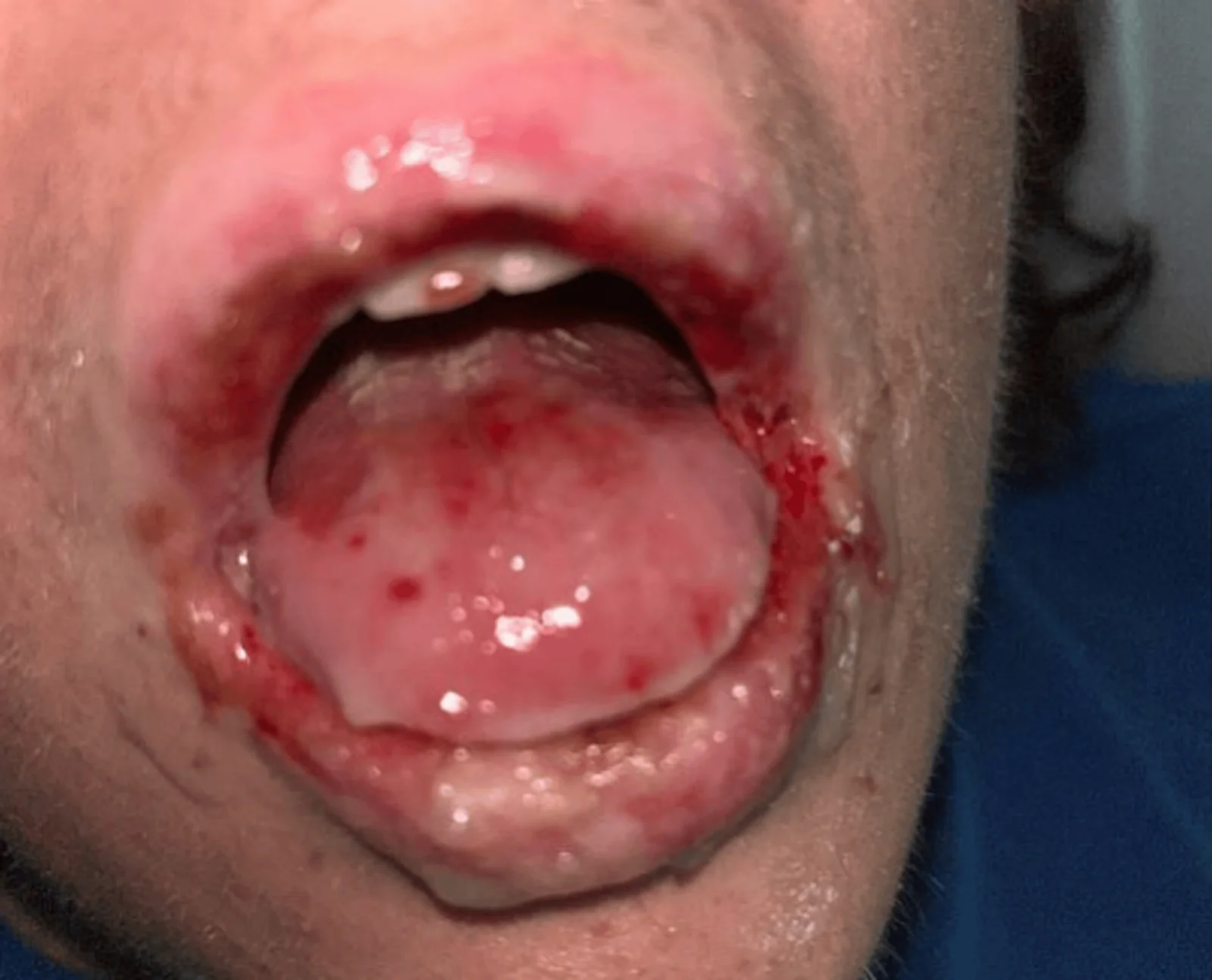
Hemorrhagic lip crusting and oral mucosal involvement on day 3 of admission

**Figure 3:**
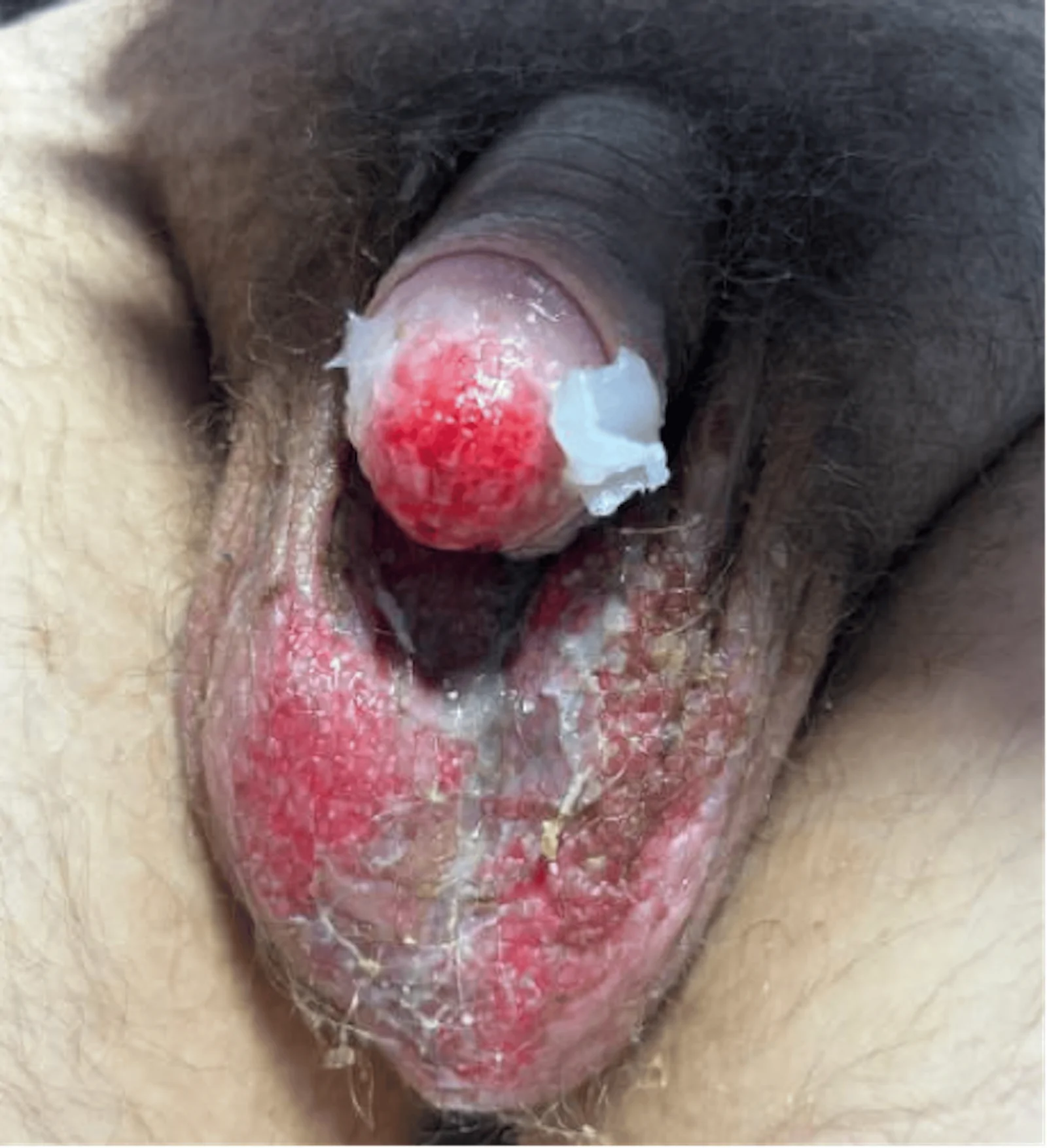
Penile mucosal involvement and scrotal skin sloughing on day 5 of admission

**Figure 4:**
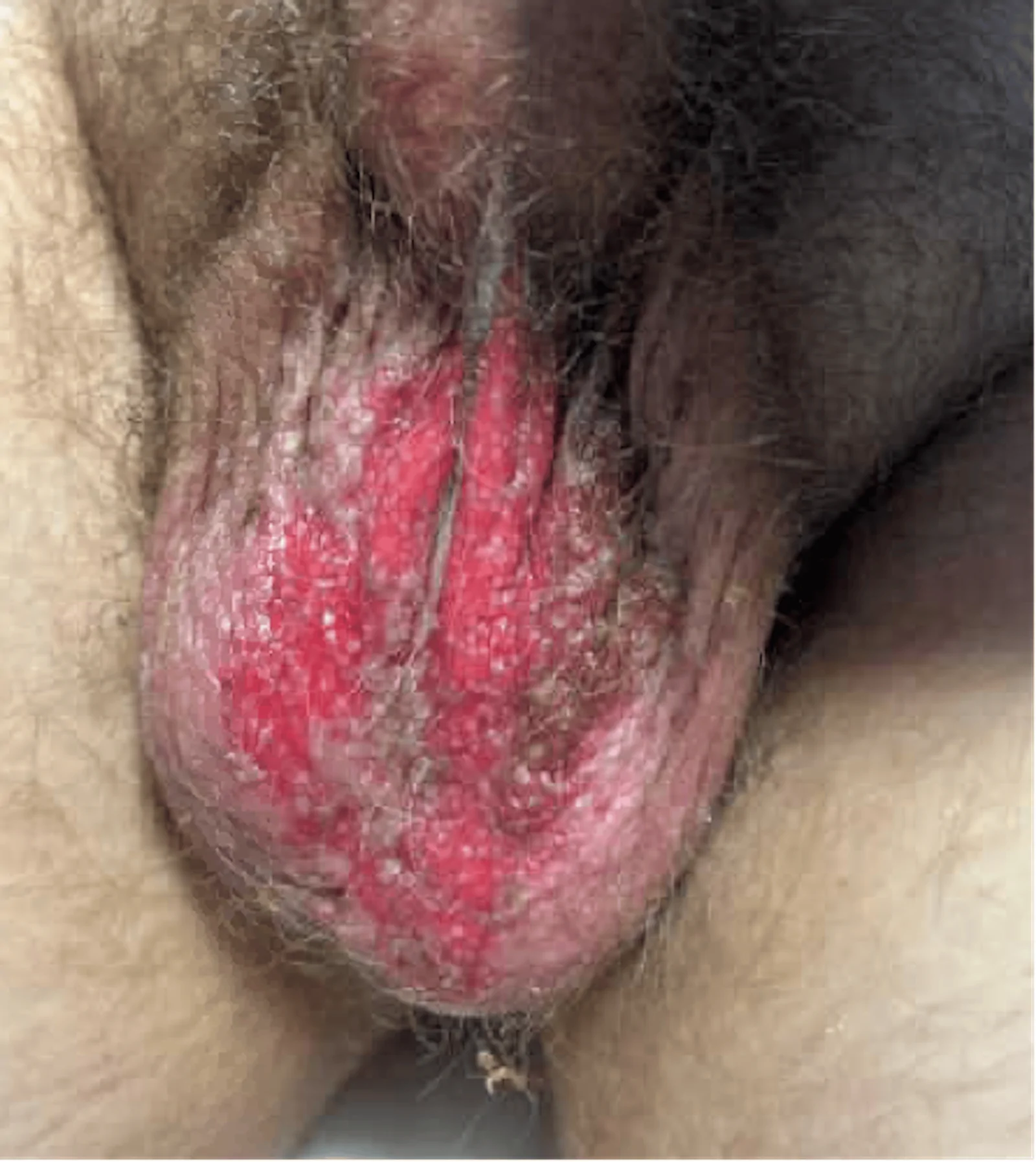
Scrotal skin sloughing on day 5 of admission with wound care

Suspecting a *Mycoplasma*-induced mucocutaneous eruption, the patient was initiated on azithromycin, accompanied by *Mycoplasma* PCR and antibody testing. The patient demonstrated significant improvement within several days. *Mycoplasma pneumoniae* IgG titers of 657 U/mL confirmed the diagnosis, while PCR results and *Mycoplasma pneumoniae* IgM titers returned negative, suggesting a possible post-infectious reaction. The patient finished a five-day azithromycin and a 10-day clindamycin course. By the time of discharge, the patient's fever had resolved, and he had shown substantial clinical improvement.

## Discussion

This case underscores the diagnostic complexity of RIME, particularly against the backdrop of a post-influenza B scenario complicated by secondary bacterial pneumonia and a subsequent *Mycoplasma*-induced mucocutaneous eruption. Initially mimicking common inflammatory and infectious conditions, the elevated CRP and ESR levels indicated systemic inflammation, with a significant rise in B-type natriuretic peptide (BNP) suggesting potential cardiac involvement-a noted concern in multisystem inflammatory syndrome but less common in RIME [4]. Elevated D-dimer and fibrinogen levels pointed towards an inflammatory state with potential coagulopathy, common in systemic infections and inflammatory reactions [3-5]. The focal lobar pneumonia led to the initial administration of clindamycin under the presumption of *Streptococcus pneumoniae* infection. 

RIME emerges with distinct features that set it apart from Stevens-Johnson syndrome/toxic epidermal necrolysis (SJS/TEN) and erythema multiforme (EM). Unlike SJS/TEN, characterized by rapidly progressing skin necrosis often triggered by drugs, and EM, known for its hallmark target papules, RIME predominantly affects mucous membranes rather than the skin [3]. RIME is characterized by a more significant impact on mucous membranes than the skin, with skin lesions being uncommon (observed in 47% of cases) or absent (34%) and seldom widespread [3,6]. This distinction is crucial in clinically evaluating and diagnosing patients presenting with mucocutaneous symptoms. The eventual diagnosis of RIME, supported by serological evidence of *Mycoplasma pneumoniae* infection, the patient's presentation of acute mucosal lesions following a respiratory prodrome, and the lack of recent high-risk medication exposure, strongly supports a diagnosis of RIME [6]. This aligns with the established understanding that in pediatric cases exhibiting acute-onset mucosal eruptions following respiratory symptoms without a history of significant drug intake, RIME emerges as the most probable diagnosis [7].

The patient's progression from typical respiratory infection symptoms to severe mucocutaneous involvement exemplifies the unpredictable nature of post-viral syndromes [8]. The complexity of this case was further amplified by multisystem involvement, which necessitated considerations of Kawasaki disease and MIS-C, underscoring the importance of a comprehensive diagnostic workup to determine the causative agent [6-8]. The elevated *Mycoplasma pneumoniae* IgG level was compatible with recent or previous exposure, but negative IgM and PCR testing and the absence of paired serology prevented the confirmation of acute infection. *Mycoplasma pneumoniae* titers and direct PCR evidence illustrate the limitations of PCR in confirming such infections, suggesting a post-infectious or immune-mediated process [8]. Despite negative *Mycoplasma* PCR results, the significant improvement following azithromycin treatment underscores the critical role of clinical judgment, using physical exam findings and history features, in managing complex cases.

## Conclusions

This case illustrates a severe mucosa-predominant eruption consistent with RIME following a respiratory illness in an adolescent. Although an elevated *Mycoplasma pneumoniae* IgG level was identified, negative PCR and IgM testing and the absence of paired serology prevented definitive attribution to acute *Mycoplasma pneumoniae* infection. The earlier positive influenza B result remained a plausible alternative trigger. The case highlights the importance of integrating the clinical phenotype, medication timeline, molecular testing, and serology before assigning a specific infectious cause. Early multidisciplinary management, including hydration, pain control, nutritional support, ophthalmologic evaluation, and targeted oral and genitourinary care, is essential.

## References

[1] Meyer Sauteur PM, Theiler M, Buettcher M, Seiler M, Weibel L, Berger C (2020). Frequency and clinical presentation of mucocutaneous disease due to Mycoplasma pneumoniae infection in children with community-acquired pneumonia. JAMA Dermatol.

[2] Song A, Nicholson C, Maguiness S (2021). Recurrent reactive infectious mucocutaneous eruption (RIME) in two adolescents triggered by several distinct pathogens including SARS-CoV-2 and influenza A. Pediatr Dermatol.

[3] Canavan TN, Mathes EF, Frieden I, Shinkai K (2015). Mycoplasma pneumoniae-induced rash and mucositis as a syndrome distinct from Stevens-Johnson syndrome and erythema multiforme: a systematic review. J Am Acad Dermatol.

[4] Ramcharan T, Nolan O, Lai CY (2020). Paediatric inflammatory multisystem syndrome: temporally associated with SARS-CoV-2 (PIMS-TS): cardiac features, management and short-term outcomes at a UK tertiary paediatric hospital. Pediatr Cardiol.

[5] Giannis D, Ziogas IA, Gianni P (2020). Coagulation disorders in coronavirus infected patients: COVID-19, SARS-CoV-1, MERS-CoV and lessons from the past. J Clin Virol.

[6] Tonkin R, Ladha M, Johnson N (2022). Reactive infectious mucocutaneous eruption - repeat etanercept after intravenous immunoglobulin: a case report. SAGE Open Med Case Rep.

[7] Ramien ML, Bruckner AL (2020). Mucocutaneous eruptions in acutely ill pediatric patients-think of Mycoplasma pneumoniae (and other infections) first. JAMA Dermatol.

[8] Santos RP, Silva M, Vieira AP, Brito C (2017). Mycoplasma pneumoniae-induced rash and mucositis: a recently described entity. BMJ Case Rep.

